# Transfection of hypoxia-inducible factor-1α mRNA upregulates the expression of genes encoding angiogenic growth factors

**DOI:** 10.1038/s41598-024-54941-w

**Published:** 2024-03-20

**Authors:** Jakub Wlodarczyk, Albert Leng, Sanaz Nourmohammadi Abadchi, Niloufar Shababi, Farzad Mokhtari-Esbuie, Shayan Gheshlaghi, Mohsen Rouhani Ravari, Emma K. Pippenger, Ali Afrasiabi, Jinny Ha, John M. Abraham, John W. Harmon

**Affiliations:** 1 Department of Surgery, Johns Hopkins University School of Medicine, 4940 Eastern Avenue, 1550 Orleans Street, Baltimore, MD 21224 USA; 2 Division of Thoracic Surgery, Department of Surgery, Johns Hopkins University School of Medicine, Baltimore, MD 21224 USA; 3 Present Address: Department of General and Oncological Surgery, Medical University of Lodz, Lodz, Poland; 4 Present Address: Department of Surgery, University of Chicago Medicine, Chicago, IL 60637 USA

**Keywords:** HIF-1a, mRNA transfection, Pedicle flap, Angiogenic genes, Drug development, Preclinical research

## Abstract

Hypoxia-Inducible Factor-1α (HIF-1α) has presented a new direction for ischemic preconditioning of surgical flaps to promote their survival. In a previous study, we demonstrated the effectiveness of HIF-1a DNA plasmids in this application. In this study, to avoid complications associated with plasmid use, we sought to express HIF-1α through mRNA transfection and determine its biological activity by measuring the upregulation of downstream angiogenic genes. We transfected six different HIF-1a mRNAs–one predominant, three variant, and two novel mutant isoforms–into primary human dermal fibroblasts using Lipofectamine, and assessed mRNA levels using RT-qPCR. At all time points examined after transfection (3, 6, and 10 h), the levels of HIF-1α transcript were significantly higher in all HIF-1α transfected cells relative to the control (all p < 0.05, unpaired Student’s T-test). Importantly, the expression of HIF-1α transcription response genes (VEGF, ANG-1, PGF, FLT1, and EDN1) was significantly higher in the cells transfected with all isoforms than with the control at six and/or ten hours post-transfection. All isoforms were transfected successfully into human fibroblast cells, resulting in the rapid upregulation of all five downstream angiogenic targets tested. These findings support the potential use of HIF-1α mRNA for protecting ischemic dermal flaps.

## Introduction

Wounds are a substantial source of suffering, healthcare costs, and morbidity in the United States and the world. Those arising from burns, trauma, and surgeries are a significant part of this burden^1,2^. Ischemic necrosis often afflicts pedicle flaps, used in the reconstruction of major wounds which have been treated by surgical debridement^3,4^. Pedicle flaps are involved in surgical procedures such as abdominal hernia repair, breast reconstruction, and plastic surgical procedures including abdominoplasties and facelifts, and can be vulnerable to surgical flap necrosis^5–10^. Necrosis also accounts for the high 35% failure rate in lower limb minor amputations^11^. For decades, researchers have searched for an effective and practical method to promote the survival of these flaps^12–15^.

What Odland wrote in 1995 remains true today in that “surgical delay is the only clinical means of improving the survival of skin flap tissue”^14^. Various methods are in use and they all seek to produce temporary ischemia in the flap, in order to induce angiogenesis and the other mechanisms of survival endogenously, before transference of the flap^12–15^. The flap is raised to produce a moderate level of ischemia. For example, a bilateral pedicle flap may be raised while still attached at both ends, so that it has two sources of blood supply, then converted into a unilateral flap for the reconstruction. A single medical alternative would be preferable to the surgical approaches currently in use.

**Figure 1:**
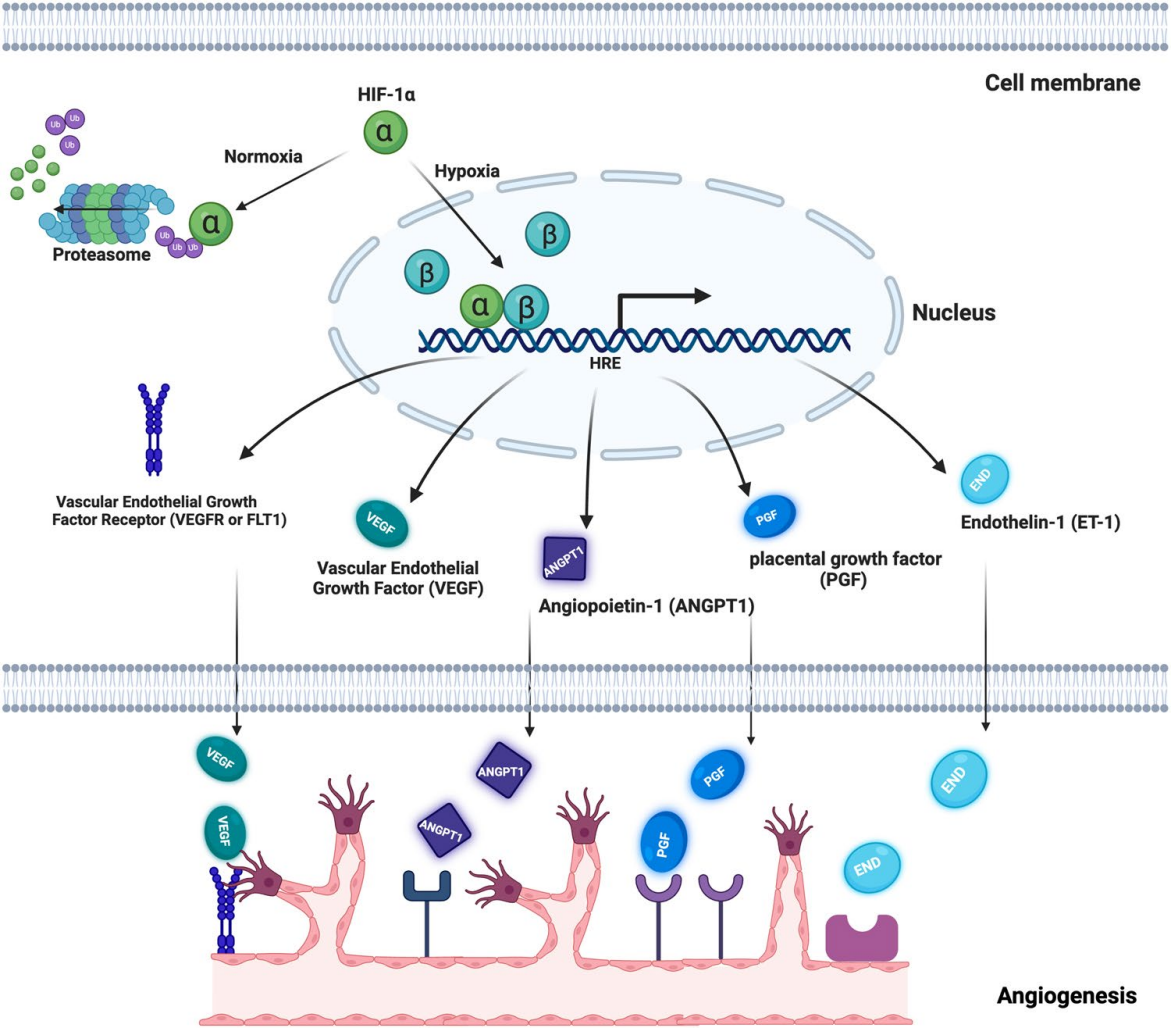
Schematic representation of HIF-1-mediated gene regulation under hypoxia. HIF-1 is a heterodimeric transcription factor composed of an O_2_-regulated HIF-1α subunit and a constitutively expressed HIF-1β subunit. Under hypoxic conditions, HIF-1α is stabilized and binds to hypoxia response elements (HREs) in the promoter regions of target genes, leading to their upregulation. HIF-1 mediates the expression of hundreds of genes, including those involved in angiogenesis.

The discovery of Hypoxia-Inducible Factor-1α (HIF-1α) has suggested a new approach to the ischemic preconditioning of surgical flaps^16,17^. HIF-1α is a subunit of a heterodimeric transcription factor, hypoxia-inducible factor 1 (HIF-1), which coordinates the response of the cells to hypoxia and thus promotes cell survival^18,19^. HIF-1α enables the cellular adjustment to hypoxic conditions through the activation of particular genes associated with vascularization, angiogenesis, metabolism, and cell survival^20^. HIF-1α has also been shown to play an important role in the maintenance of natural killer cells as well as other cells of innate immunity^21,22^. It regulates the expression of more than 200 genes that encode angiogenic growth factors, including vascular endothelial growth factor (VEGF), placental growth factor (PGF), angiopoietins (ANGPT1, ANGPT2), stromal cell-derived factor-1(SDF-1), and platelet-derived growth factor B (PDGF-B)^23,24^ (Fig. 1). Many of these growth factors are relatively unstable in vivo and have a short half-life^25,26^. Gene therapy may ensure continuous production of therapeutic products by addressing both the lability of HIF-1α and the short half-life of its response-gene transcription products.

Several studies have explored the potential role of HIF-1α in wound healing and in the ischemic preconditioning of surgical flaps^27–29^. Du et al. showed that HIF-1α gene transfection improves burn wound healing in aged mice^29^. Takaku et al. provided support for this concept by demonstrating that elevated levels of HIF-1α could promote tissue survival in a mouse model^28^. Prior experiences with DNA plasmid delivery demonstrated that a time-limited and localized delivery could potentially be achieved suitable for the clinical applications^29,30^. As such, surgical pedicle flap survival in the rat model was improved by pre-treating the flaps with HIF-1α using a DNA expression plasmid vector^31^. However, this approach to HIF-1α delivery involved two serious drawbacks. First, the plasmid is transported to and integrated into the cell nucleus, which by definition results in a genomic alteration. Second, DNA expression plasmids often use a constitutive promoter to express the encoded gene indefinitely, which can have harmful long-term consequences.

This paper describes the use of HIF-1α mRNA transcripts to express HIF-1α in order to avoid these two complications. First, the mRNA is translated in the cytoplasm and never reaches the nucleus. Second, mRNA has a defined half-life, meaning that the HIF-1α transcript will degrade at a steady, known rate. Thus, we avoid the problem of indefinite HIF-1α overexpression. If more of the drug is needed, it can simply be injected again at a specified dose. The recent development and use of mRNA to deliver COVID-19 vaccines amply demonstrated the potential of this emerging technology and the possibility for various clinical applications^32,33^.

The use of mRNA transcripts could produce a therapeutic with the potential to be produced in a fast, relatively inexpensive, and straightforward manner. The current study introduces the expression of HIF-1α through mRNA transfection into primary dermal fibroblast cells and to determine its biological activity, by measuring the upregulation of downstream angiogenic genes. The generation and delivery of biologically active HIF-1α could potentially provide a powerful therapeutic for promoting the survival of surgical pedicle flaps.

## Methods

### Isolation, cloning, and production in vitro of HIF-1α mRNA isoforms

DNA PCR primers corresponding to the 5' and 3' ends of the HIF-1α transcript were obtained (IDT, Coralville, Iowa). The 5’ primers contained a 20-base T7 RNA polymerase promoter sequence. Using a leukocyte cDNA library purchased from TaKaRa Corporation (TaKaRa Bio, San Jose, CA), PCR reactions were performed using an Avantage2 Taq polymerase APCR kit from TaKaRa. Various cycles were performed and the PCR products were monitored for expected bands and the absence of contaminating products of the wrong size by visualizing on an agarose gel. PCR products were cloned using a TA TOPO cloning kit (ThermoFisher, Waltham, MA). Individual clone DNAs were isolated (Qiagen, Baltimore, MD), digested with restriction enzymes, and run on agarose gels. Clones displaying different restriction enzyme digestion patterns were identified, and the plasmid inserts were Sanger sequenced at the JHU Sequencing Core Facility. This sequencing identified a predominant HIF-1α form (P1) and three transcriptional variants (V1, V2, and V3). The HiScribe T7 ARCA mRNA kit (NEB, Ipswich, MA) yielded full-length transcripts with a 5’-methyl cap and a 3’ poly-A tail. Each mRNA reaction was purified by an RNA Cleanup kit (NEB, Ipswich, MA), quantitated, and visualized on an agarose gel.

### Synthesis of two novel isoforms via mutation of two prolines

The predominant full-length HIF-1α isoform contains two proline residues known to participate in degradation. V1 and V2 each contain one of these prolines, and V3 contains both of these proline residues. The GeneArt™ Site-Directed Mutagenesis PLUS System was used to eliminate these prolines from the predominant isoform (P1) in order to test their ability to increase HIF-1α response gene levels. Theoretically, the removal of these prolines would increase the protein half-life, although it would not render the protein completely resistant to degradation and turnover. Oligo primers were designed to change one nucleotide in a particular sequence using PCR amplification. The use of in vitro DNA recombination yielded a circular plasmid used to transform competent E. coli cells. Individual clones were selected, and the plasmid DNA was extracted and sequenced at the JHU facility to verify that the correct directed mutations had occurred. At positions 402 and 564, the Prolines were changed to Alanine in mutants P2 and P3.

### Cell-culture

Primary human dermal fibroblasts were obtained from the American Type Culture Collection (ATCC, Manassas, VA) and were cultured using a fibroblast growth medium purchased from the manufacturer. When the flasks reached 80% confluency, the cells were split using phosphate-buffered saline (PBS) and trypsin and then resuspended in the medium. The cells were incubated in a humidified atmosphere of 5% CO_2_ at 37 °C and the medium was replaced every 2–3 days.

### Transfection of fibroblasts with HIF-1α mRNA transcripts

Transfection of the fibroblasts with the HIF-1α mRNA transcripts was done with Lipofectamine MessengerMAX Transfection Reagent (ThermoFisher, Waltham, MA), according to instructions provided by the manufacturer. An extensive series of experiments was carried out to determine the optimal dosage of lipofectamine reagent and the varying amounts of HIF-1α mRNA required for maximum increase in response genes mRNA levels. These experiments are not shown. Primary human fibroblasts were transfected with different isoforms of HIF-1α mRNA transcripts in triplicate, and a 96-well cell culture plate was used to seed each well with 10 000 primary dermal fibroblasts. The transfection was performed using 0.3 μg of the appropriate mRNA and 0.3 ul of lipofectamine in each well in 24 h. The cells were harvested at 3-, 6-, and 10-h time points post-transfection, and total RNA was extracted.

### Quantitation of response genes

The medium in the cell culture plate wells was removed and the wells were rinsed with Tris-buffered saline (Sigma-Aldrich, Saint Louis, MO) at various times post-transfection. According to instructions provided by the manufacturer, Buffer RLT from the RNeasy mini-kit (Qiagen, Baltimore, MD) was then directly added to each well to lyse the cells and harvest RNA. Real-time quantitative RT PCR was performed to determine response genes expression levels using selected gene-specific primers (IDT, Coralville, Iowa) and a QuantiNova SYBR Green Probe RT-PCR kit (Qiagen, Baltimore, MD) using a CFX96 Real-Time PCR Detection System (Bio-Rad, Hercules, CA). The response genes were: VEGF, PGF, ANGPT1, FMS-related receptor tyrosine kinase 1 (FLT1), and, Endothelin 1 (EDN1). To ensure accurate comparisons between samples, all specific gene quantitative values were normalized to the expression level of the housekeeping gene β-actin.

### Statistical analysis

To compare the differences in gene expression levels between different isoforms, the Student’s t-test was used. The significance level (alpha) was set at 0.05 for all statistical tests, and p-values less than 0.05, 0.01, or 0.001 are designated in the figures.

## Results

### Isolation of isoforms and generation of mRNA

**Figure 2:**
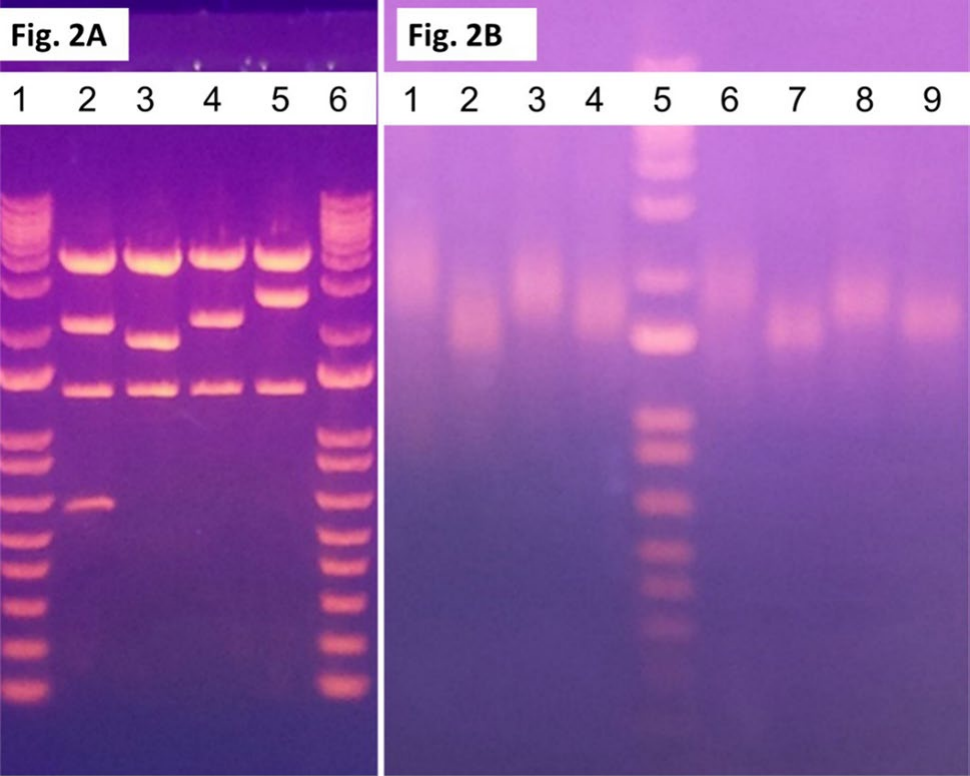
Isolation, Cloning, and Production In Vitro of Four HIF-1α mRNA Transcript Isoforms. (**A**) lanes show the isolation of a full-length “predominant’ form as well as three transcript variants. Sanger DNA sequencing covered 100% of each HIF-1α isoform. The full-length predominant form contained an open reading frame of 826 amino acids, while variants 1, 2, and 3 contained 424, 516, and 785 amino acids respectively. The HiScribe T7 ARCA mRNA kit (NEB, Ipswich, MA) yielded transcripts with a 5’-methyl-cap and a 3’ poly-A tail. Each mRNA reaction was purified using RNA Cleanup (NEB, Ipswich, MA), quantitated, and visualized on an agarose gel. (**B**) lanes on the left represent the samples before purification; lanes on the right represent the samples after purification.

Individual colonies were selected, grown overnight in LB with 100 ug/ml of ampicillin, and the plasmid DNA was extracted and quantitated. The clones were digested with EcoRI restriction enzyme and run on a 1% agarose gel (Fig. 2A). The predominant P1 isoform and three variant isoforms (V1, V2, V3) were linearized and mRNA was generated in vitro as described above. These mRNA products were run on a non-denaturing agarose gel to confirm the successful mRNA synthesis and the subsequent clean-up of the mRNAs (Fig. 2B) (Supplementary Fig. 1). The DNA sequence of each clone was determined by DNA sequencing.

### Structural characterization of isoforms and mutated isoforms

**Figure 3:**
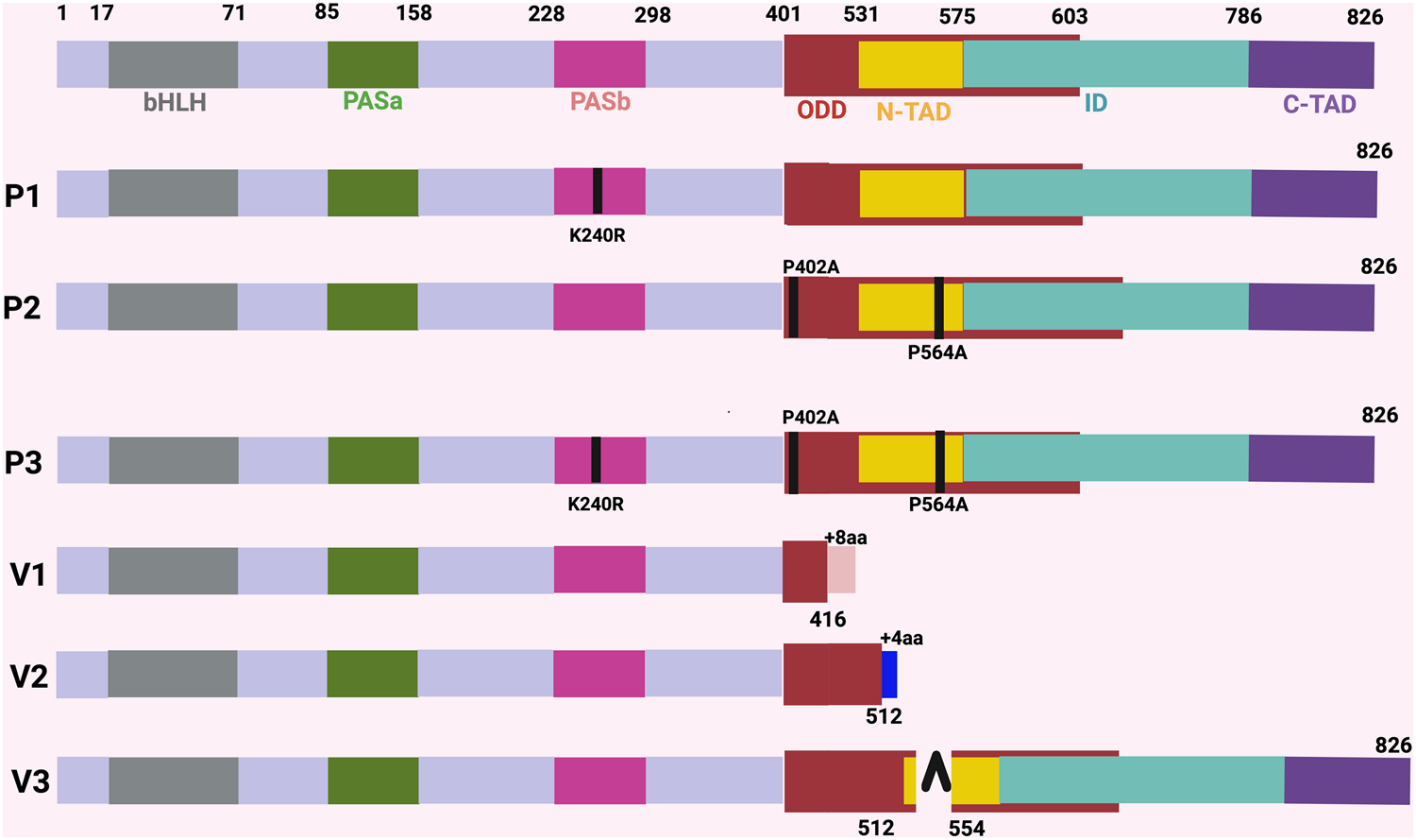
Structural motifs and domains of HIF-1α protein and six isoforms and mutants. HIF-1α mRNAs that encode the full-length protein (P1, P2, P3) and the three shortened splice variants (V1, V2, V3) are shown. P402A and P564A represent Prolines involved in HIF-1α degradation. bHLH is basic helix-loop-helix, PASa (Per-Arnt-Sim) and PASb are molecular sensors, ODD is oxygen-dependent degradation, ID is an inhibitory domain, N-TAD, and C-TAD are amino- and carboxy-transactivation domains.

The structural description of the predominant isoform (P1), the three variant isoforms (V1, V2, V3), and the two mutated isoforms of P1 (P2, P3) are shown in Fig. 3**.** Each of these mRNAs is described in detail as follows:

P1 is the full-length predominant version of HIF-1α with 826 amino acids (in NCBI Reference Sequence NM_001530). It contains Guanosine at nucleotide 1011 instead of adenosine, which changes the Reference Sequence from Lysine to Arginine. It also has two prolines (Proline 402, Proline 564) that are involved in HIF-1α degradation via the VHL-mediated ubiquitin protease pathway.

P2 has the same characteristics as P1, but with the Arginine at position 240 (nucleotide 1011) changed to the Reference Sequence Lysine. Additionally, the two Prolines (Proline 402, Proline 564) that are involved in HIF-1α degradation have been changed to alternate amino acids.

P3 has the same characteristics as P1, but retains the Arginine at nucleotide 1011and has the two degradation Prolines changed to alternate amino acids.

V1 is a naturally-occurring isoform that has a major deletion and contains only 424 amino acids, and possesses only one of the two Prolines reported to have a role in degradation.

V2 is a naturally-occurring isoform that contains 516 amino acids and has only one of the two Prolines reported to have a role in degradation.

V3 is a naturally-occurring isoform that contains 785 amino acids and has both of the Prolines reported to have a role in degradation.

### Transfection analysis

**Figure 4:**
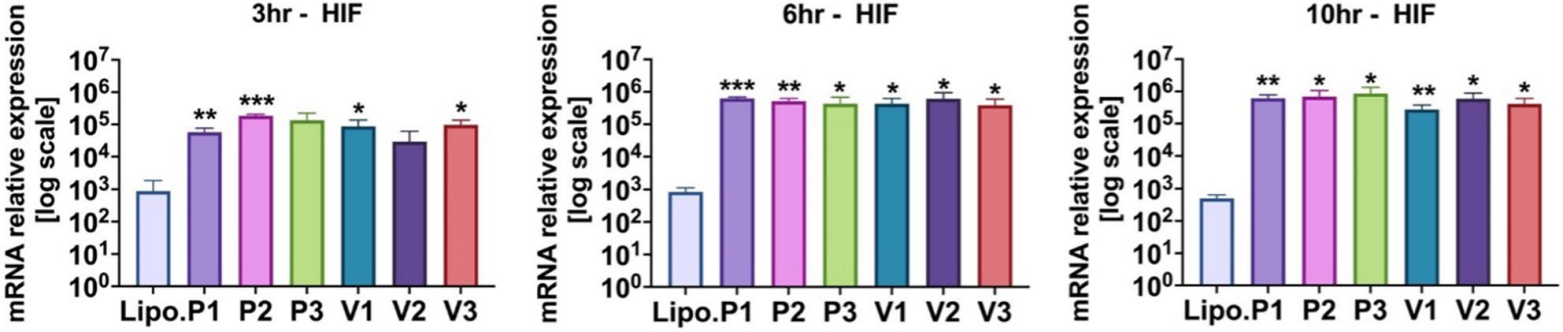
HIF-1α mRNAs (P1, P2, P3. V1, V2, V3) were transfected into primary dermal fibroblasts. At 3, 6, or 10 h, total RNA was extracted and assayed for HIF-1α expression levels by qRT-PCR. Values were normalized to beta-actin levels. “Lipo.” is lipofectamine alone control. The Student’s T-test was used to determine significance and the mean and standard deviation of independent triplicate wells is shown. *p = 0.05 or lower, **p = 0.01 or lower, and ***p = 0.001 or lower.

Transfection of HIF-1α mRNA into primary dermal fibroblasts led to changes in the expression levels of various genes involved in angiogenesis. As seen in Fig. 4, HIF-1α mRNA levels increased significantly at all time points examined (3, 6, and 10 h post-transfection) when compared to the control group that was treated with Lipofectamine only (all p < 0.05).

**Figure 5:**
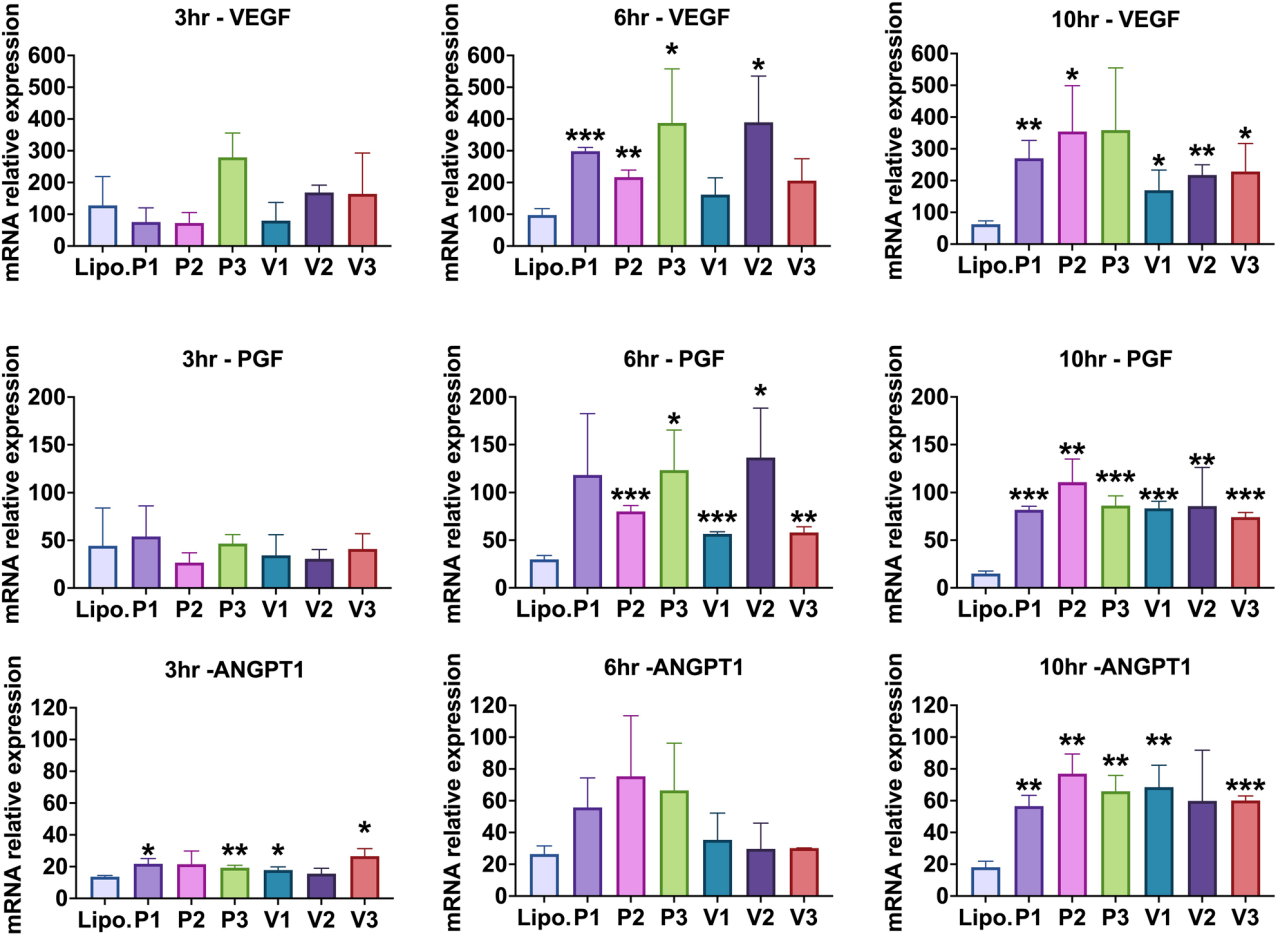
HIF-1α mRNAs (P1, P2, P3. V1, V2, V3) were transfected into primary dermal fibroblasts. At 3, 6, or 10 h, total RNA was extracted and assayed for VEGF, PGF, or ANGPT1, expression levels by qRT-PCR. Values were normalized to beta-actin levels. “Lipo.” is lipofectamine alone control. The Student’s T-test was used to determine significance and the mean and standard deviation of independent triplicate wells is shown. *p = 0.05 or lower, **p = 0.01 or lower, and ***p = 0.001 or lower.

**Figure 6:**
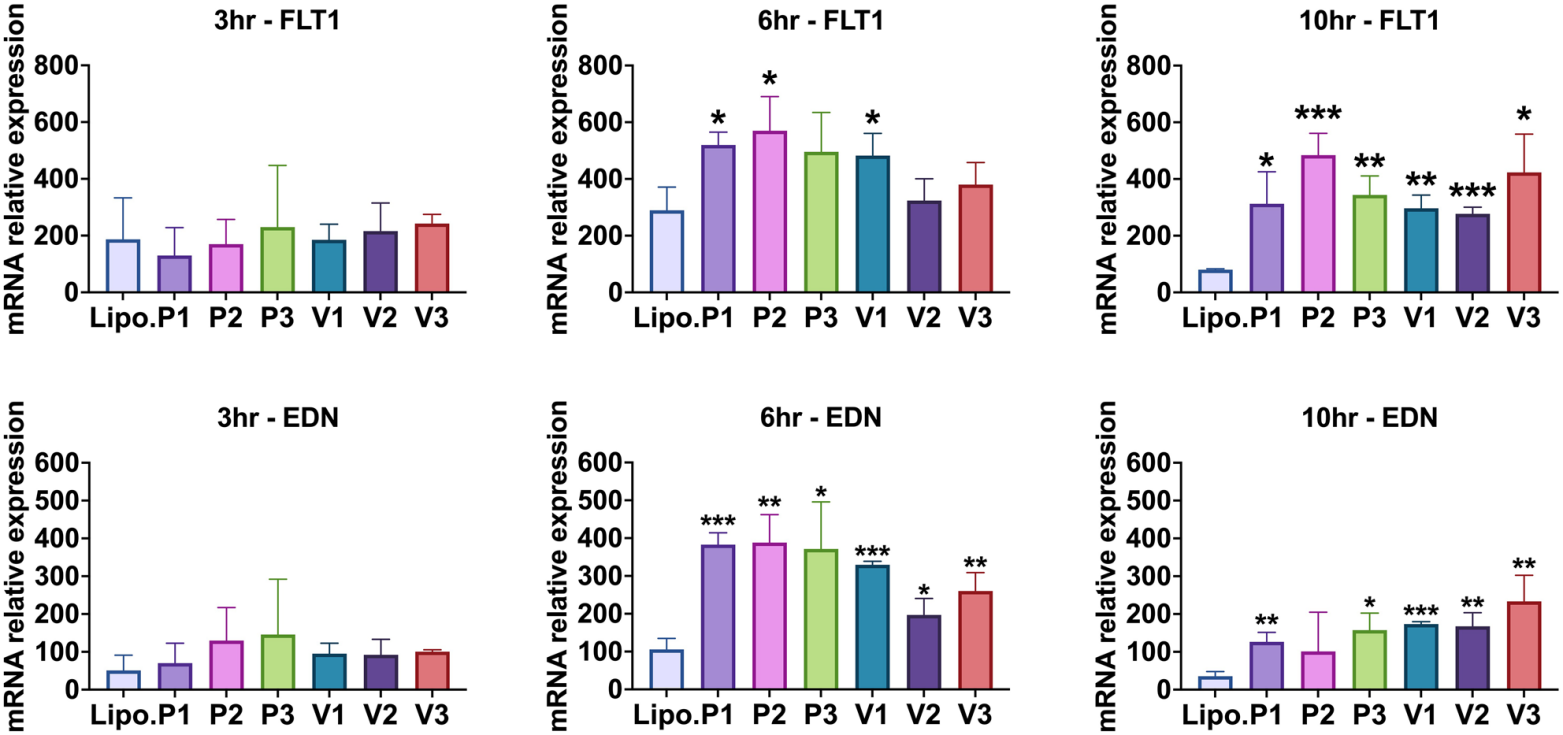
HIF-1α mRNAs (P1, P2, P3. V1, V2, V3) were transfected into primary dermal fibroblasts. At 3, 6, or 10 h, total RNA was extracted and assayed for FLT-1, or EDN1 expression levels by qRT-PCR. Values were normalized to beta-actin levels. “Lipo.” is lipofectamine alone control. The Student’s T-test was used to determine significance and the mean and standard deviation of independent triplicate wells is shown. *p = 0.05 or lower, **p = 0.01 or lower, and ***p = 0.001 or lower.

Figures 5 and 6 show that at 6 and 10 h post-transfection, all groups treated with the isoforms and mutated isoforms showed a significant increase in VEGF transcript levels compared to the control group except for isoforms V1 and V3 at 6 h post-transfection (all p < 0.05), and isoform P3 at 10 h post-transfection (all p < 0.05). However, at 3 h post-transfection, no treatment groups had a significant change in VEGF mRNA expression when compared to the control group.

For PGF mRNA levels, at 6 h post-transfection, all groups treated with the isoforms and mutated isoforms showed a significant increase in PGF transcript levels compared to the control group except for isoform P1 (all p < 0.05). At 10 h post-transfection, all groups treated with the isoforms showed a very significant increase when compared to the control group (all p < 0.05). However, at 3 h post-transfection, no treatment groups showed a significant difference compared to the control group.

At 3 h post-transfection, all groups treated with the isoforms and mutated isoforms showed a significant increase in ANGPT1 hours post-transfection except for P2 and V2. At 10 h post-transfection, all treatment groups showed a significant increase in ANGPT1 transcripts compared to the control group except for isoform V2 (all p < 0.05).

For FLT1 mRNA levels, at 6 h post-transfection, the groups treated with P1, P2, and V1 showed a significant increase in FLT1 transcripts compared to the control group (all p < 0.05). At 10 h post-transfection, all treatment groups showed a significant increase in FLT1 mRNA compared to the control group (all p < 0.05). At 3 h post-transfection, no treatment groups showed a significant change compared to the control group.

Regarding EDN1 mRNA levels, at 6 h post-transfection, all groups treated with the isoforms showed a significant increase in expression when compared to the control group (all p < 0.05). At 10 h post-transfection, all treatment groups also showed a significant increase in EDN1 expression when compared to the control group except for isoform P2 (all p < 0.05).

Overall, intracellular HIF-1α transcript levels increased significantly within a 3 h time frame for all transfected HIF-1α isoforms and remained consistently elevated for at least 10 h. All the downstream angiogenic genes had a response that generally peaked at 6 h after initial HIF-1α transfection.

## Discussion

For decades researchers have searched for an effective and practical method to promote the survival of surgical pedicle flaps^12,13,34^. Various forms of surgical delay are used to precondition flaps to survive ischemia. These methods seek to produce temporary ischemia in the flap and thus to provoke angiogenesis and other mechanisms of survival endogenously prior to mobilizing the flap for reconstruction. This approach is an invasive surgical strategy and carries its own set of risks. Thus, there is a need for a non-invasive medical alternative^14,15^.

Several non-invasive alternatives have been explored with conflicting results. Recently, Luo et al. evaluated the application of fibroblasts transfected with modified mRNA encoding the stromal cell derived factor-1α in rats, which resulted in reduction of tissue necrosis and promotion of neovascularization in random skin flaps^35^. Other approach, involving the direct application of angiogenic growth factors to improve angiogenesis, has been tested in several clinical trials which have reported minimal success^36^. These attempts have relied on the use of a single protein or combinations of up to three proteins, such as VEGF, PDGF, TGFβ, ANGPT1, and other growth factors, to improve overall wound repair^37–39^. Interest has turned to gene therapy, since it may be possible to facilitate sustained production of angiogenic products by altering gene expression^40–42^. Shams et al. demonstrated that fibroblasts transfected with recombinant VEGF plasmid enhanced angiogenesis in vitro and in vivo. This study showed that, in the early stages of the healing process, VEGF-expressing human foreskin fibroblast cells attached to a scaffold of polyurethane-cellulose acetate (PU-CA) enhanced vascular lumen formation relative to similar fibroblast cells which lack the VEGF-expressing plasmid. However, these results were not statistically significant^43^. In contrast to these two approaches of merely expressing a single downstream gene like VEGF or FGF, or providing the final products of those genes, we favor a continuous production of angiogenic products by taking advantage of a master gene at the beginning of the cascade of angiogenesis. This orchestrates changes in gene expression in sequence with biologically relevant kinetics—potentially a much more powerful and effective approach.

Hypoxia-Inducible Factor-1α (HIF-1α), a master regulator of the adaptive response to hypoxia, has presented a new direction for ischemic preconditioning of surgical flaps and promoting their survival^18,28^. Because HIF-1a regulates the expression of hundreds of growth factors such as VEGF, PGF, ANGPT1, ANGPT2, SDF-1, PDGF-B, etc., as well as various kinds of cytokines and enzymes that promote tissue viability under ischemic conditions, it could be promising as a therapeutic for ischemic preconditioning of surgical flaps^28,44^. Takaku et al. have demonstrated that tissue survival in a mouse model is promoted by elevating levels of HIF-1α^28^. They created elevated levels of HIF-1α via systemic treatment with dimethyloxalylglycine (DMOG)^29^. This sets the stage for later studies which investigated the efficacy of utilizing a DNA expression plasmid vector to deliver HIF-1α. This approach seeks to avoid the systemic elevation of HIF-1α levels concomitant with DMOG treatment while mitigating the well-known lability of HIF-1α and its angiogenic and pro-proliferative transcription factors^27,31,45^. Yamamoto et al. investigated the RNA-binding protein LIN28A and showed that LIN28A upregulates HIF-1 α expression via mRNA stabilization. Authors confirmed that the increased level of HIF-1 α promoted angiogenesis in vivo^46^.

In one such study, Kelly et al. performed an adenovirus-mediated HIF-1αCA5 (AdCA5) plasmid transduction, twenty-four hours after subretinal injection, but observed only a transient induction of HIF-1α mRNA^45^. Our own prior experience with DNA plasmid delivery suggested that it would be possible to achieve a steady and localized delivery, aided at times by plasmid transduction techniques such as electroporation, suitable for the clinical application of the therapy^27,29,30^. Our group showed that intradermal injection of HIF-1a plasmid in wounded diabetic mice followed by electroporation resulted in increased levels of HIF-1α mRNA at the injection site on day 3 and increased levels of VEGF, PLGF, PDGF-B, and ANGPT2 mRNA on day 7 post-injection^27^.

While DNA expression plasmids solve the issue of the short half-life of angiogenic proteins, they may integrate into host cell nuclear DNA and express the encoded gene indefinitely, which can be harmful. To overcome this limitation, we engineered a system that potentially transfers biologically active HIF-1α mRNA into the wound environment. This approach utilizes therapeutic mRNA transfer techniques currently used in cancer and COVID-19 vaccines. HIF-1a mRNA transfected into cells does not travel to the nucleus, allowing for fast translation and a predictable decline of the transcript over time^32,33,40,47,48^.

The establishment of in vitro transcription (IVT) and mRNA chemical modification has resolved many of mRNA’s limitations, including innate low stability and possible immunogenic issues while retaining their translation efficiency^40,49–55^. Besides its two transactivation domains, HIF-1a also possesses an oxygen-dependent degradation (ODD) domain whose two proline residues are vulnerable to hydroxylation by proline-hydroxylase-2 (PHD2)^49^. Hydroxylation in turn allows for the binding of the Von Hippel-Lindau (VHL) protein, which finally targets the HIF-1a subunit for proteasomal degradation^49,50^. Iron chelators and inhibitors of the prolyl hydroxylase domain (PHD) enzyme are among the most widely used chemical agents to stabilize HIF-1a and induce a hypoxic response, even under normoxic conditions^49,56^. In a few other studies, small molecules which target VHL and the ubiquitination of HIF-1a have also been evaluated^51–53^. In our study, by contrast, the HIF-1α molecule was modified to delete these two prolines and thereby increase its resistance to prolyl hydroxylases that function to destroy HIF-1α.

Dong et al. showed that modified VEGFA mRNA delivered by ionizable lipid nanoparticles can be taken up and expressed effectively by endothelial cells in vitro*.* They also showed that the VEGFA mRNA-LNP was able to significantly promote angiogenesis, tissue regeneration, and wound healing in vivo^57^. This is consistent with our results in which our modified full-length predominant HIF-1α isoform (P1) and its three splice-variants isoforms (V1, V2, V3) were shown to be biologically active in vitro (Fig. 5). Additionally, we tested two site-directed mutant isoforms (P2, P3). These each contained mutations encoding for proline, which is susceptible to hydroxylation under conditions of normoxia. Consequently, these isoforms were able to impair HIF1-a ubiquitin-dependent degradation. We demonstrated that these mutant isoforms will not negatively affect HIF-1α transcriptional activity and that they are biologically active. When we introduced each of the six HIF-1α mRNA isoforms into dermal fibroblasts, our results at six and ten hours post-transfection showed a rapid and regulated, yet transient expression of HIF-1α response genes (Fig. 5).

Many different materials have been used for mRNA delivery, such as lipids, lipid-like materials, polymers and protein derivatives^47^. Lipid nanoparticles have been successfully employed for delivering small molecules, siRNA drugs, and mRNA. These lipid-based carriers are capable of enhancing the stability of synthetic mRNA in vivo and facilitating its delivery to the target cells^47,57,58^. We used lipofectamine, a liposomal cationic lipid-based carrier, to transfect primary human dermal fibroblast cells with our therapeutic HIF-1a mRNA isoforms. Moreover, increasing interest in mRNA delivery and rapidly growing number of studies in this area, where novel and more efficiency systems are proposed, suggest that in the closest future we will be able to even more precisely use them in HIF and other mRNA transfections^59,60^.

In conclusion, our results show the successful transfection of all six HIF-1α mRNA isoforms into human primary fibroblast cells. As a result, we observed the upregulation of all five downstream angiogenic targets tested in a rapid and regulated manner. In future surgical situations, the HIF-1α mRNA could be injected locally into and around the reconstruction site. If additional doses are required, these could be administered as needed. However, more studies must be done to test each of these six isoforms and mutants in an animal model to determine if any or all are able to elicit an increase in important downstream factors in vivo in the wound environment. Additional studies will compare the transfection efficiency of our therapeutic composition of HIF-1α mRNA formulated in lipofectamine, already tested in vitro, against a new delivery system involving Polypeptide Lipid Nanoparticles (PLNP)^45,61^.

### Supplementary Information

Supplementary Figure 1.

## Data Availability

Nucleotide sequences have been uploaded to NCBI GenBank and are available with following accession numbers: BankIt2759727 SeqP1 OR762216, BankIt2759727 SeqP2 OR762217, BankIt2759727 SeqP3 OR762218, BankIt2759727 SeqV1 OR762219, BankIt2759727 SeqV2 OR762220, BankIt2759727 SeqV3 OR762221.
