# Supplementary material for: Transfection of hypoxia-inducible factor-1α mRNA upregulates the expression of genes encoding angiogenic growth factors
Source: Sci Rep. 2024 Mar 20;14:6738. doi: 10.1038/s41598-024-54941-w (PMC10954730; doi:10.1038/s41598-024-54941-w)

Supp. Fig. 1A. This is the original unaltered and unedited entire gel picture showing different loading quantities in order to optimize the part of the gel chosen for the figure.


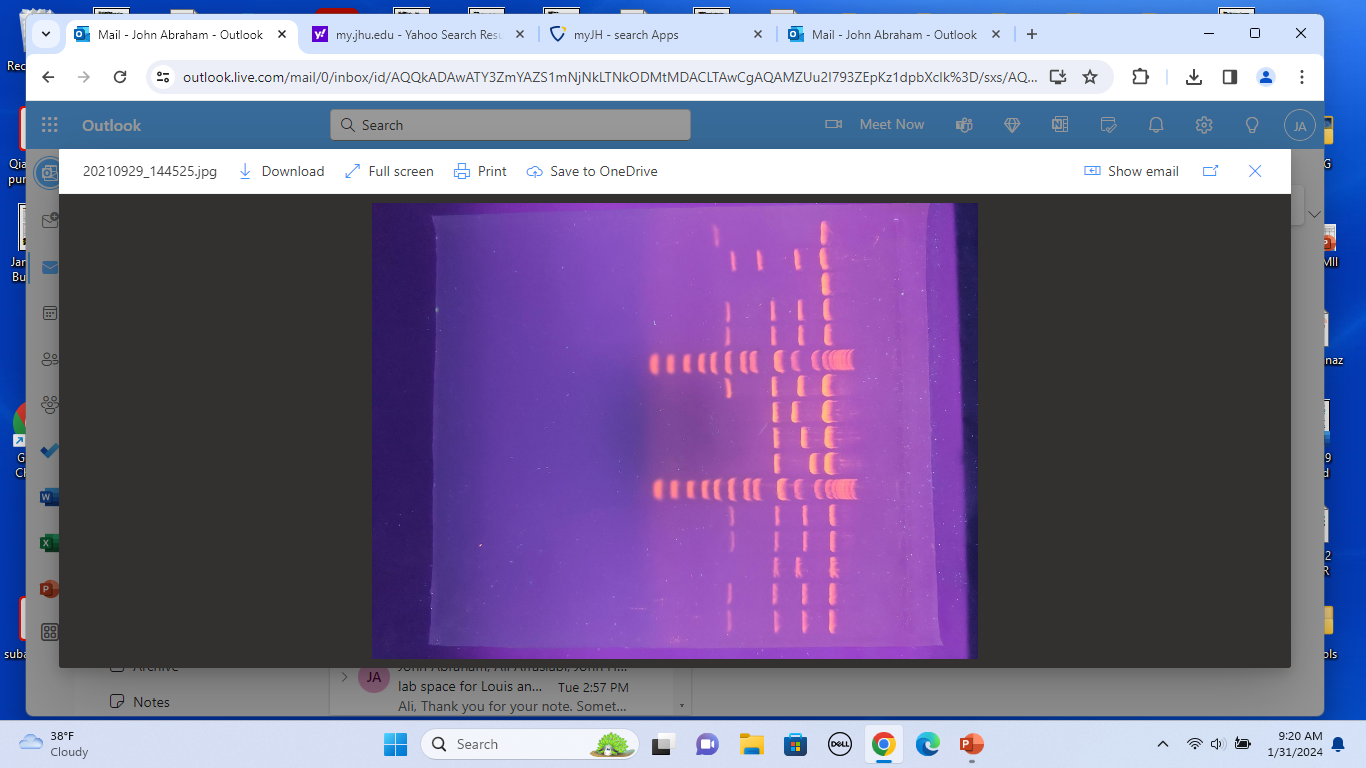


Supp. Fig. 1B. This is the original entire unaltered and unedited gel picture showing different isoform mRNAs.


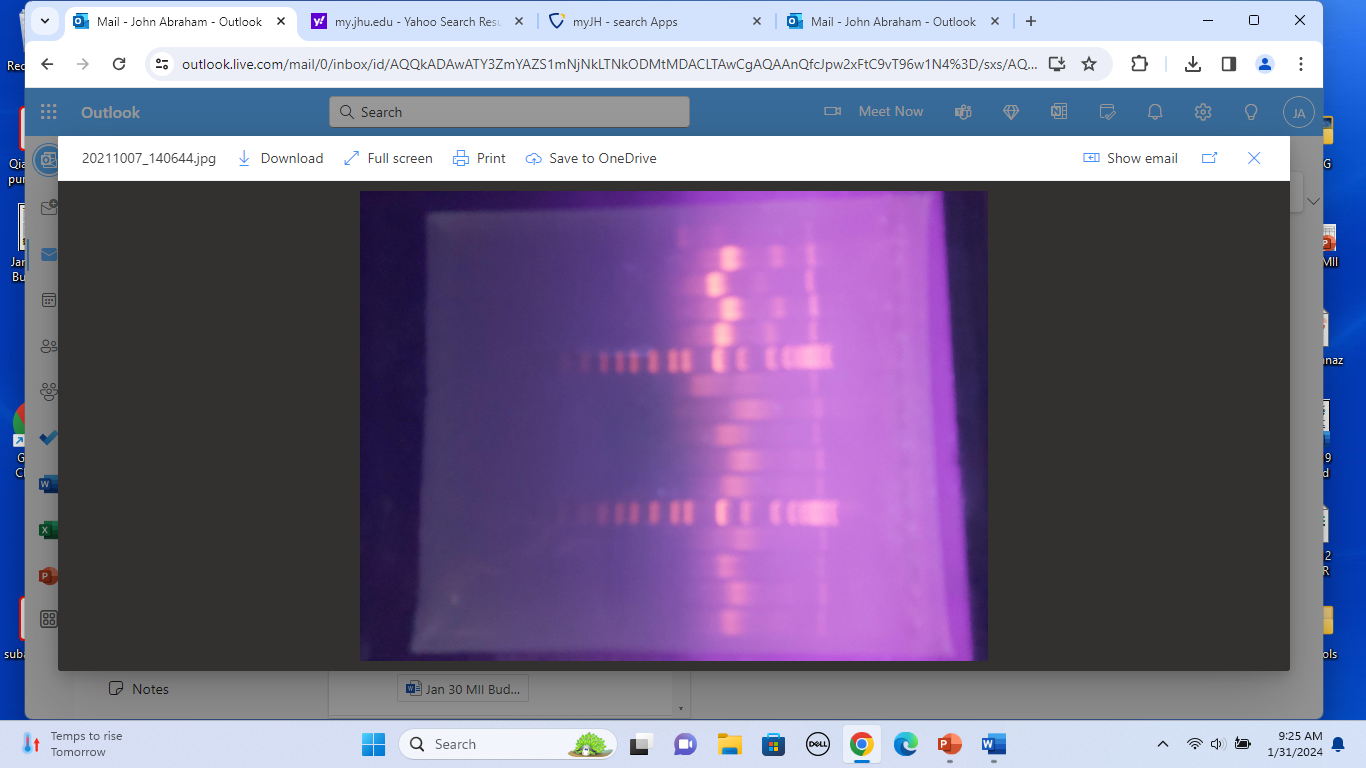

Supplement: Supplementary file 1 — Supplementary Figure 1. [file 41598_2024_54941_MOESM1_ESM.docx]
